# Process evaluation of a whole-of-community systems approach to address childhood obesity in western Victoria, Australia

**DOI:** 10.1186/s12889-020-08576-x

**Published:** 2020-04-06

**Authors:** Ebony Jenkins, Janette Lowe, Steven Allender, Kristy A. Bolton

**Affiliations:** 1 Southern Grampians Glenelg Primary Care Partnership, Hamilton, Australia; 2 Global Obesity Centre, Institute for Health Transformation, Deakin University, Geelong, Australia

**Keywords:** Process evaluation, Systems thinking, Collective impact, Obesity prevention, Community, Asset based community development

## Abstract

**Background:**

SEA Change Portland is a systems-based approach implemented in Portland, Victoria that utilises local community resources to sustainably prevent and reduce the prevalence of childhood obesity. Action is implemented by community-led task teams with differing priority areas, and supported by a steering committee representative of four collaborating organisations. This study examines the SEA Change Portland process to identify significant events, enablers and barriers of its development and implementation to date as reported by key stakeholders involved in implementation during the first 12 months.

**Methods:**

Semi-structured interviews were conducted with eight steering group members and three community task team members. Data was collected utilising open ended interview questions to gather in-depth information regarding program implementation, and the individual attitudes, beliefs and experiences of key stakeholders.

**Results:**

Data were analysed under three key themes: collective impact, systems thinking and asset based community development (ABCD). Participants gave perceptions of significant events; factors positively and negatively affecting the process; reasons for becoming involved in the process; perceived efficacy of task teams, principles of diversity and areas of concern. Themes emerged from participant responses allowing were categorisation of their responses into four key process stages: initial lead up; process development; establishing community ownership of the obesity system; and community action.

**Conclusion:**

Collective impact was a crucial element in applying the systems thinking. Strong and equitable relationships between steering organisations and topic experts provided the initiative with a sustainable foundation, and ABCD promotes community ownership and future sustainability. Understanding the process of implementing a new whole-of-community systems approach to childhood obesity prevention such as SEA Change Portland has provided vital knowledge for other communities regarding enablers and barriers of this promising approach.

## Background

Childhood obesity is a great public health concern with 27% Australian children classified as overweight/obese [1]; and increased risk of developing cardiovascular disease, type 2 diabetes, musculoskeletal disorders and some cancers [2]. Key modifiable determinants of childhood obesity include feeding practices in early years, diet and physical activity [3]. Monitoring data collected across the region; which included the regional town, Portland, in the Glenelg Shire, Victoria, Australia; revealed higher than expected rates of children to be overweight or obese in some years by gender compared to the national average [4]. For example, 32% of girls in year 4 in government and independent schools in the region were classified as overweight or obese [4].

Obesity is a complex problem requiring intervention across multiple levels of society. Addressing obesity requires active consideration of the causes and therefore possible solutions that are driven by interactions between several components of a system, governed by feedback, and are non-linear and unpredictable [5]. The multifaceted nature of obesity requires a multi-strategy, community-led and participatory intervention that mobilises community assets across all levels of a system to illicit sustainable solutions [6]. Due to the depth and variety of key driving influences on obesity, traditional health promotion approaches are often not the best fit as they are without the advantages for dealing with complexity as are provided by systems science [7].

Systems thinking involves the incorporation of individual, ecological, social and political factors, as well as interactions between each of these domains [8]. A systems approach analyses meaning attached to actions and power relations between actors, assisting projects to obtain a more sustainable reach [8]. More recently, community action to solve complex problems has applied the collective impact framework which seeks among other things to bring organisations together to focus upon a common agenda in aim of facilitating sustainable change [9]. Key aspects of collective impact include a common agenda and shared understanding of the problem, shared accountability, mutual plans of action, shared measurement systems and continuous communication and information sharing [10].

Sustainable Eating Activity Change Portland (SEA Change Portland) is a systems-based approach implemented in Portland that utilises local community resources and connects stakeholders for collaboration with the aim of delivering sustained change of weight status in children aged 7–12 years, through implementation of community generated and led changes to environments in which children live learn and play. SEA Change represents the pilot community in a larger stepped wedge cluster randomized trial of capacity building for systems thinking in community-based obesity prevention [11]. A systems map co-created by the community identifies the systemic causes of childhood obesity in their community [12]; and represents the grounded logic for model for their action. Co-ordinated action is implemented by community-led task teams with differing priority areas supported by a steering group. Community task team objectives include but are not limited to; increasing healthy eating in schools, increasing physical activity, increasing active transport, increasing water consumption and decreasing unhealthy local marketing, sponsorship and fundraising. More information around the implementation of SEA Change Portland can be found on the webpage, https://seachangeportland.com.au. Along with application of the collective impact framework, efforts to connect and mobilise the community to act are made utilising asset based community development (ABCD), a strategy promoting sustainable community development. ABCD is driven by 3 key principles; 1) shifting mindsets from needs and deficiencies to assets and capabilities; 2) mapping and connecting a diverse range of community assets; and, 3) harnessing community assets for action [13].

Currently information regarding effectiveness of systems approaches addressing childhood obesity are scarce. Whilst there are few community-led systems-based obesity prevention initiatives, there is a gap in documented process evaluations of these efforts. Undertaking an investigation of process can assist in understanding experiences of stakeholders, assess whether project development and delivery has been as intended and identify modifications in delivery as required [14, 15].

This study presents an evaluation of the SEA Change Portland process in order to identify significant events, enablers and barriers of its development and implementation from commencement to the first 12 months of implementation as reported by the key stakeholders involved in implementation of this initiative.

## Methods

This cross-sectional study adheres to the STROBE checklist guidelines (Supplementary Table 1).

### Setting

The regional seaside town of Portland is the main centre of the Glenelg Local Government Area and is home to approximately 9700 residents [16]. Portland is located in the south west of Victoria, Australia, 360 km from the states capital city Melbourne.

### Participants

A purposive sample of key informants were recruited according to their knowledge of the SEA Change Portland process, drawn from agency stakeholders and community task groups. A minimum of one member of each of the four collaborating steering group organisations was interviewed. Recommendations were sought from steering group members as to which task team and community contributors should be approached for data collection.

Steering group members interviewed included the following: two health promotion officers from the local health service; the Executive Officer and three project officers from the Primary Care Partnership; the Active Communities manager from the local shire council and the healthy lifestyles/tobacco action worker from the Aboriginal health corporation. Task team members and community contributors included one volunteer who coordinates a cooking program within a local primary school; one local radio presenter who was also a current member of the Australian Breastfeeding Association; and, the principal of a local primary school.

### Interview process

A focus group was facilitated within an existing steering group meeting, as this is proven to encourage active participation and have ability to strengthen and empower the existing network [17]. Semi-structured interviews were conducted, utilising open ended interview questions to gather in-depth information regarding program implementation so far, and the individual attitudes, beliefs and experiences of key stakeholders. These questions were designed specifically for the purpose of this study. Interview responses were recorded through note taking. There were seven questions and it took approximately 45 min to complete an interview. At the conclusion of each interview, the interviewer repeated the noted responses to the participant to confirm the accuracy of the information recorded. Of the 16 potential informants approached, 11 agreed to participate (69% participation rate).

### Interview questions

The following interview questions were asked of steering group members, with no deviations from wording of the questions nor a change in the order of questions;

- What have been the most significant events/processes of SEA Change Portland so far?
- What factors have positively affected the SEA Change Portland process so far?
- What factors have negatively affected the SEA Change Portland process so far?
- Why have people/organisations become involved with SEA Change Portland?
- What are the factors influencing task team efficiency?
- Have processes sufficiently encompassed principles of diversity?
- Specific areas of concern regarding how the process has occurred so far or the direction in which SEA Change Portland is heading into the future

The following interview questions were asked of community task team members, with no deviations from wording of the questions nor a change in the order of questions;

- What encouraged you to become involved with SEA Change Portland?
- From your perspective, what events/processes in SEA Change Portland have been the most significant?
- What factors have positively affected your involvement in the SEA Change Portland process so far?
- What factors have negatively affected your involvement in the SEA Change Portland process so far?
- How has SEA Change Portland enabled you to make change?
- Have processes sufficiently encompasses principles of diversity?
- Specific areas of concern regarding how the process has occurred so far or the direction in which SEA Change Portland is heading into the future

Note that the questions above are the full, definitive list of questions used in this study.

### Analysis

A thematic analysis of participant responses to interview questions was undertaken by the interviewer. A key stakeholder who helped guide the development of the interview questions and was involved in the work and community context provided intellectual input into the thematic analysis conducted by the interviewer. The analysis was guided by a pragmatic approach where the interviewer was learning from process data for application into practice.

Interview responses identified have been categorised according to the level at which they reflect the three key frameworks informing SEA Change Portland; systems thinking, collective impact and asset based community development (ABCD) and recommendations were then categorised under four key process stages which emerged during analysis; initial lead up, process development, establishing community ownership of the obesity system and community action.

#### Ethical approval

This study was approved by Deakin University Human Ethics Advisory Group (HEAG-H 118_2017).

#### Trial registration

SEA Change Portland is one community involved in a larger stepped wedge cluster randomized trial of capacity building for systems thinking in community-based obesity prevention [11] which was retrospectively registered 26/07/2016 Australian New Zealand Clinical Trials Registry (ACTRN12616000980437). https://www.anzctr.org.au/Trial/Registration/TrialReview.aspx?id=371109&isReview=true

## Results

Participant responses regarding their perceptions and experiences of the SEA Change Portland process were thematically categorised into systems thinking, collective impact and ABCD; and are presented in Table 1.

**Table 1:** Evaluation of the SEA Change Portland Process according to steering group and community task team members

	**Systems thinking**	**Collective impact**	**ABCD**
**Significant events in process**	• Group model building/systems mapping workshops • The ‘organic evolution’ of SEA Change Portland across all areas and levels of the system	• Information sharing within the steering group via online platform (Zoho) • Engaging Department of Health • Formation of relationship with Deakin University and topic experts • Contribution to development and adherence to applicable Municipal Public Health and Wellbeing Plan • Commitment of varied local agencies to participation in a steering group collaborative • Regular steering group meetings • Steering group action planning	• ABCD workshop with steering group and community leaders • Connecting people • Formation of community task teams • Steering group action planning
**Positive factors impacting process**	• Defining the problem with the community through group model building/ systems mapping workshops • Having a diverse steering group promoting direct community links	• Strong organisational and personal steering group relationships • Leverage of topic experts (Deakin University, The Collaboration for Impact and Peter Kenyan) • Trust and support between collaborating agencies • Equitable steering group dynamic	• Strong community engagement and recruitment • Social media presence (radio program) • ABCD workshops – the ABCD approach as a new and different approach to engaging community • Health promotion workers having existing strong relationships with community members
**Negative factors impacting process**	• Poor community engagement at some levels of the system – attributed to limited interest, capacity, time and miscommunication of the approach. • Limited diversity in attendance at community group model building/ systems mapping workshops • Lack of referral throughout the process to community created resources, e.g. the systems map	• Miscommunication and confusion over perception of ownership within steering group organisations • Misaligning thought processes of steering group members • A lack of support from within steering group agencies given to those working at ground level • Targeted engagement efforts – efforts could have been less focused upon agency and more on community change agents over all levels of the system Process of voting on the SEA Change Portland name was dominated by larger steering group organisations without wider consultation – steering group members voted with their respective agencies • Communication issues in development of terms of reference and media policy	• Timing of the ABCD workshop – this could’ve been more effective had it occurred earlier, in conjunction with the systems mapping workshops • Systems mapping workshop content could have been more ‘action orientated’, rather than solely focused on problem definition • Systems mapping workshop content was too heavy for some community members present with low health literacy, no additional efforts were undertaken to engage these groups through a different process
**Triggers for involvement in process**	• Development of a briefing paper and systems map highlighting a complex problem • Understanding of the problem for the system as a whole • Identifying a new approach towards obesity prevention is required	• Through personal and organisational relationships • Understanding of the value in collective impact	• Providing community members specific prompts to action • ABCD workshops • Facebook, webpage and radio program • Identifying opportunity for asset based involvement • Positive influence of community members already engaged
**Specific areas of concern regarding the process**	• True alignment with systems thinking – the community systems map is not being used effectively	• Disproportional access to knowledge and resources between the steering group, organisations and individuals • Inappropriate information sharing missing the opportunity to enhance community engagement	• Inconsistent media representation of SEA Change Portland • Community ownership of the program is not yet at the desired level • Has community capacity been built enough for ‘doing’?

*ABCD* Asset based community development

### Significant events in SEA change Portland

Throughout the evolution of SEA Change Portland a number of events have been identified to have significant impact upon development and progression of the initiative. The processes identified in Table 1 were the ‘stand outs’ of SEA Change Portland and hold most crucial significance of all events throughout the process thus far according to the steering group and community task team members interviewed.

### Factors affecting the SEA change Portland process

Processes contributing to implementation of SEA Change Portland can be mostly attributed to the influence of collective impact in tackling the complex issue of obesity. Table 1 reveals what participants view to be the most enabling factors to success of SEA Change Portland to date. A number of areas for improvement were also identified.

### Triggers for involvement in the SEA change Portland process

Table 1 reveals both participants reported triggers to their personal involvement in the initiative and perceived prompts for the involvement of others. The use of group model building or systems mapping was an important step in the community collectively unpacking the complexity of obesity in the local context and identifying their capacity to become involved. Through establishing this shared understanding, community members became familiar with and started to the value parts in which each contributor could play a vital role. This new recognition of importance of collaboration catalysed the involvement of many additional community members.

### Specific areas of concern in the SEA change Portland process

Although general thoughts regarding the future of SEA Change Portland are extremely positive, there were some concerns identified within interviews towards future direction. Participant concerns for future direction of the initiative are categorised in Table 1 below.

### Task team efficacy

Table 2 reveals participant responses when asked to comment on perceived factors influencing efficiency of developed task teams. Whilst it was broadly acknowledged any kind of support or contribution is valuable to a task team, respondents confidently enforced the importance of gathering passionate community with diverse skillsets to maintain momentum and achieve timely success.

**Table 2:** Perceived factors influencing efficiency of task teams according to steering group and community task team members in SEA Change Portland

Factors increasing efficiency of task teams	Factors decreasing efficiency of task teams
Networking and connecting the ‘right’ community members for task teams	Community task teams led by health professionals, moving away from ABCD.
Engaging those with personal interest	Lack of interest
Engaging those with passion for broader community development, not just health	Time constraints
Engaging those with a high level of readiness to act	
Presence of at least one ‘key driver’ within each task team	
Support from all four collaborating agencies (steering group)	

### SEA change Portland and principles of diversity

Table 3 summarises participant responses when asked to comment on to what degree they believe SEA Change Portland encompasses and considers diversity. Promoting diversity within SEA Change Portland has been a challenge for the steering group, as although a number of diverse population groups were initially consulted to be involved in the process, several marginalised groups have not had the capacity to become involved and are therefore absent in representation.

**Table 3:** Perceived factors influencing diversity of SEA Change Portland according to steering group and community task team members

Factors encouraging diversity	Factors discouraging diversity
Systems map identified determinants of obesity for all population groups at all levels of the system	Aim of most processes and recruitment at the ‘middle class’
Steering group deliberately directs vast effort into engaging all population groups	The key focus of children has deterred engagement of some groups including the disability, mental health and elderly sectors
	Engagement and sequence of engaging community members to become initiative leaders
	Steering group has not yet engaged marginalised groups whom at the outset had a null or low response to the process
	Group model building/ systems mapping and ABCD workshops had capped numbers

## Discussion

This study aimed to evaluate perspectives of key stakeholders involved in implementing the process of SEA Change Portland, a systems-based approach addressing childhood obesity, in the first 12 months of the initiative. Responses from the steering group and community task team members revealed valuable information regarding their perception of significant events; factors positively and negatively affecting the process; why they have become involved in the process; perceived efficacy of task teams and principles of diversity and areas of concern.

This study highlights strong reciprocal relationships between collective impact, systems thinking and ABCD approaches in the implementation of SEA Change Portland. Use of all three concepts appear, at least in the short term, to prevent the initiative from reverting back to ‘business as usual’ (considered by practitioners as a less desirable way of working deemed to produce less sustainable outcomes). For this community, collective impact was a crucial element to be imbedded in a community-based systems approach to obesity prevention. Strong and equitable relationships between steering organisations and topic experts are vital and provide the initiative with a sustainable foundation, before ABCD is introduced to promote community ownership of the approach and further its sustainability into the future.

To establish community ownership of the system, a diverse range of community members should collectively unpack the complexity of the problem and its key influences. Group model building, or systems mapping, was used in SEA Change Portland to assist this process. It’s desirable that all community sectors are engaged in this process, and participants are not selected according to influence or authority, rather the social assets they provide. Any materials co-created by community should be referred to regularly to reinforce community ownership.

### Implications for future practice

**Fig. 1:**
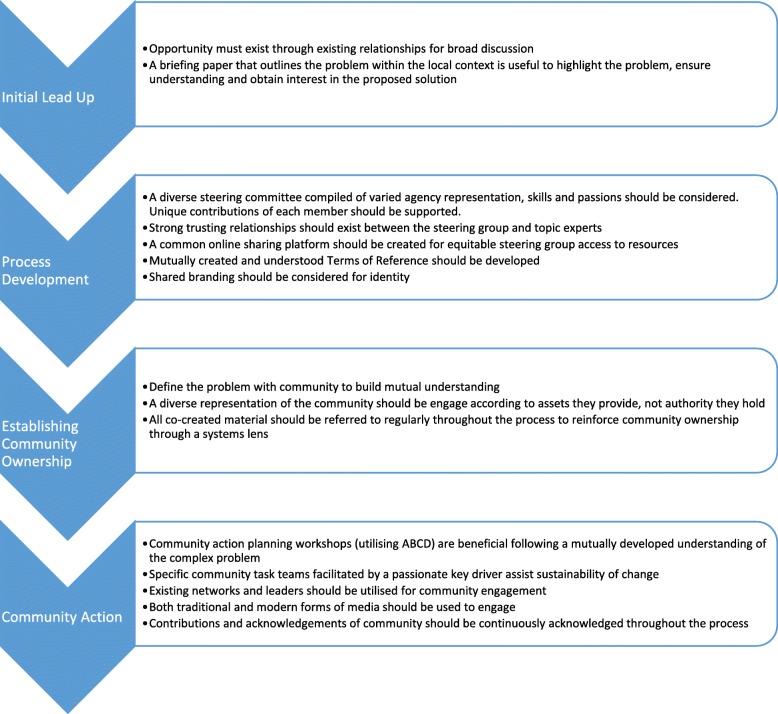
Recommendations from SEA Change Portland for future consideration

Communities that seek to apply systems thinking methods underpinned by a collective impact framework may benefit from the lessons learned in application of same in SEA Change Portland. Recommendations based upon participant responses were made under four key process stages: initial lead up, process development, establishing community ownership of the obesity system and community action (Fig. 1). Any future community-led systems-based obesity prevention efforts should consider these recommendations in addition to documenting and publishing their learnings to build the evidence base for the implementation process of these initiatives. Future research should capture the view of those who do not engage in the approach and consider learnings from a systems lens in other community-based interventions, such as thinking about feedback loops and delays regarding factors influencing project implementation [18], how to apply this approach in policy making processes [19], and how to strengthen using a sustainability framework in planning, implementation and reporting stages to help communities maintain a healthy weight [20].

Figure 1 reveals key recommendations under each stage of the implementation process as generated through analyses of perceptions and experiences of SEA Change Portland stakeholders.

In utilising a combination of systems thinking, ABCD and collective impact across all four process stages, additional recommendations were identified from responses that are applicable throughout the entire process of implementation. These are as follows;

- Ensure all steering group organisations have shared understanding over ownership of the process and an appropriate level of readiness and high willingness to communicate openly as part of a collaborative
- Engage community members first through assets they provide for community action, not agencies or organisations they represent
- Utilise existing relationships between organisations, employees and community members to enhance engagement
- Ensure all population groups are initially engaged and attempts are made to re-engage those whom display a null or low response to the process
- Continue community engagement throughout the entire process to further build capacity and community ownership
- Ensure all community workshop content is relevant, useful, easily understood and promotes action
- Provide community contributors and champions specific prompts to action and allow for development of connections to enable action they wouldn’t have established otherwise
- Ensure each task team consists of people with interest in health and community development, who have capacity to contribute to the process

### Strengths and weaknesses of the study

Currently information regarding effectiveness of systems approaches addressing childhood obesity is scarce. Whilst there are few community-led systems-based obesity prevention initiatives, there is an even vaster gap in documented process evaluations of those that are in place or have been implemented in Australia previously. The strengths of this study include strong engagement of key stakeholders and participation in the systems approach. Additionally, the researcher conducting the study was embedded within the community, providing rich detail and community context which is not typically available to researchers. We do acknowledge several limitations of this study which include its small sample size and participation of multiple respondents from a single organisation - consequently steering group responses may not reflect a generalised view of the entire collaborative. However, the stakeholders recruited for this study were the most appropriate individuals to target to understand the emerging science regarding the effectiveness of community-based interventions. Due to the size of the community and known relationship between interviewer and one of the steering group organisations, social desirability bias may also be present in responses. Key stakeholders interviewed were all heavily engaged so whilst barriers to implementation are valid, note that they are from the perspective of those motivated to implement and strengthen this approach to tackling childhood obesity. Note-taking rather than recording the interviews may also potentially miss information. However noted responses were repeated to the key stakeholder to confirm it reflected their perspective accurately. Thematic analysis was conducted by one interviewer rather than multiple researchers; however in this pragmatic approach there was a joint function of learning from the process data collected and applying it in practice.

## Conclusion

The evaluation revealed collective impact as a crucial element of this initiative and the application of systems thinking within the approach. Strong and equitable relationships between steering organisations, their employees and topic experts provide the initiative with a sustainable foundation, and ABCD promotes community ownership and future sustainability. Understanding the process of implementing a new whole-of-community systems approach to childhood obesity prevention such as SEA Change Portland has provided vital knowledge for other communities regarding enablers and barriers of this promising approach.

## Supplementary information

**Additional file 1.**

## Data Availability

The datasets generated and/or analysed during the current study are not publicly available due to low sample number size which could potentially compromise participant privacy and lack of consent from participants to share data with third parties.

## Abbreviation

ABCD: Asset based community development
